# Social outbreak and its association with insomnia and daytime sleepiness in Chile

**DOI:** 10.5935/1984-0063.20210023

**Published:** 2022

**Authors:** Rafael Andrés Pizarro-Mena, Samuel Duran-Aguero, Andrés Silva

**Affiliations:** 1 Facultad de Ciencias de la Salud, Universidad San Sebastián, Los Leones, Santiago, Chile.; 2 Facultad de Ciencias para el Cuidado de la Salud, Universidad San Sebastián, Los Leones, Santiago, Chile.; 3 Universidad Central de Chile, Facultad de Economía, Gobierno y Comunicaciones, Santiago, Chile.

**Keywords:** Insomnia, Sleepiness, Riots, Social Outbreak, Sleep

## Abstract

**Objectives:**

To associate the effects of the social outbreak with insomnia and daytime sleepiness according to the distance from the riots.

**Material and Methods:**

Cross-sectional analytical study; a non-probabilistic sampling was carried out at a national level. The Google Forms tool was used; a document was submitted using a national database. The instrument consisted of four sections: socio-demographic data, biopsychosocial symptoms, insomnia severity index (ISI), and the Epworth sleepiness scale (ESS). The data were analyzed using descriptive statistics and the zero-inflated negative binomial model.

**Results:**

Of a total of 2,532 surveyed people, 29% were male; 43% was younger than 30 years old. The 50% of the sample suffers from sleepiness and 71% shows some type of insomnia. The marginal effects of the zero-inflated negative binomial model show that women, people aged 51 or older, who are neither studying a healthcare degree nor working in the healthcare sector, that are exposed to 4 or more hours per day to the news and that live in areas near or very near the riots, have significantly higher ISI (marginal effect 1.356, SE 0.381, p-value 0.000) and ESS scores (marginal effect 0.693, SE 0.320, p-value 0.030). To live/work in rioting areas has the greater marginal effect compared to other determinants. Finally, neither employment status nor educational level are associated with significant effects in the aforementioned scales.

**Conclusion:**

The riots occurred during the social outbreak of October 2019 in Chile had an effect on insomnia and daytime sleepiness. Particularly, to live/work in rioting areas has the greater marginal effect compared to other determinants.

## INTRODUCTION

From October 2019 to March 2020, Chile experienced a social crisis; the protests stopped with the arrival of the COVID-19 pandemic. This situation of violence might be encompassed within the wide concept of collective violence mentioned by the World Health Organization (WHO)^1^.

Research about the impact of collective violence on health has been characterized by a relative disciplinary fragmentation and a relatively narrow approach, with an emphasis on post-traumatic stress disorder (PTSD) or other psychiatric conditions like depression, without significantly addressing other dimensions of psychosocial health, such as sleep quality^2^. In addition, from the perspective of the psychological reaction to violent events, traumatic reactions are often accompanied by adaptive reactions, such as stupor, anxiety, irritability, despair, psychosomatic alterations, and sleep disorders^2^. In this environment, individuals may develop avoidant and self-isolation behaviors, feel a sense of guilt and embarrassment, restrict themselves to showing solidarity or suffer cognitive disconnection (affecting their attention, language, perception, among other functions) and substance abuse may occur^2,3^. It has been even described that the impact is harder when subjects are physically, temporarily and psychologically closer to the events of collective violence^3^. Thus, we begin to figure out how social movements and outbreaks are taking their toll on biopsychosocial health of people in the affected communities and having consequences that include sleep alterations.

The protests, as well as the extensive media coverage, have resulted in an increase in the prevalence of insomnia in the population^4^. Insomnia has been mentioned as a significant public health problem affecting between 10% and 15% of the overall population in the USA^5^, with similar rates of prevalence among diverse age groups in several countries^6-8^, including Chile^9,10^.

One of the consequences of insomnia is daytime sleepiness. Symptoms of insomnia include: difficulty falling asleep or staying asleep, waking up in the early morning, unsatisfactory sleep and distressed or altered daily performance due to sleep alterations^11^. People with insomnia report a significantly poorer quality of life^12^, have a higher risk of accidents^13^, and have high rates of psychiatric comorbidities^14,15^. Consequences of excessive daytime sleepiness include the inability to stay alert and awake during the day, and a more general feeling of drowsiness; its presence affects tasks that require vigilance, memory, and executive functions^16^.

Insomnia has been related to a series of specific mental health problems that include depression, PTSD, and suicidal tendencies, and problems of physical health like hypertension, diabetes, and cardiac events^17-19^.

In addition, previous studies indicate that the exposure to specific types of trauma increases the probabilities that insomnia is duplicated or tripled^20^. This finding is consistent with previous data that link insomnia with sexual trauma, physical assault and exposure to natural disasters^21,22^.

In connection with the preceding facts, it is not a surprise that insomnia and daytime sleepiness entail a significant economic burden due to the high cost of medical care and the decreased labor productivity^17^.

However, there is limited information on sleep alterations among the general population after a social outbreak without a war or natural disasters, taking into account the diversity and magnitude of the problem.

The objective of the present study is to associate the effects of the social outbreak on insomnia and daytime sleepiness in relation with the distance of the rioting areas.

## MATERIAL AND METHODS

Cross-sectional analytical study; a non-probabilistic sampling was carried out at a national level. The Google Forms tool was used; a document was submitted using a national database (n=5,000) and distributed through social networks (Facebook, Instagram and Twitter). Participants, after accepting the informed consent, answered an online questionnaire titled “*Characterization of the biopsychosocial effects on health and sleep of people after a month of the social outbreak in Chile (2019)”.* The study was developed following the declaration of Helsinki, taking into consideration the bioethical principles of research involving human beings and was submitted to the ethics committee of the *Servicio de Salud Metropolitano Sur* (South Metropolitan Health Service), which approved this study, the questionnaire, and its application. The inclusion criteria listed were: people aged 18 years or older living in continental or insular Chile, with the ability to follow written instructions, without impaired vision (or that can be corrected with orthotics) and who are able to answer the self-report *online* questionnaire. The questionnaire was available from November 28^th^ to December 30^th^.

### Questionnaire

The measuring instrument consisted of four sections with a total of 35 questions. The instrument consisted of closed questions with a single answer, closed questions with multiple choices and questions with open answers. The first section was aimed at collecting sociodemographic data (it consisted of 16 questions: gender; age; nationality; level of education; region of the country; city of origin; proximity and exposure to protests and rioting areas; frequency of riots and their consequences; media used to learn about the riots and how long people were exposed to such media). The original questionnaire includes, as separate questions: how far they live, work and shop-run errands with respect to the rioting areas. Specifically, in each question, proximity was determined based on well-known areas and measured in meters considering the logic of a radius, in 5 levels listed below: 1) “In the very same area of protests and riots (located in the same block, same street, streets intersection, adjacent or contiguous street)”, which is equivalent to a 125m radius; 2) “Within a radius of 2 to 3 blocks away from the area of protests and riots (same neighborhood)”, which is equivalent to a radius ranging from 126m to 375m; 3) “Located more than 4 to 5 blocks away from the area of protests and riots (another neighborhood)”, which is equivalent to a radius ranging from 376m to 625m or more; 4) “Very far from the area of protests and riots (another sector or commune)”; and 5) “In a sector where no protests or riots have been held”. Subsequently, respondents who chose alternatives 4 or 5 in the three questions (where they live, work, and commonly shop-run errands) were classified as “far from the rioting area”. If the respondents chose alternatives 1 and 2 in the three questions, they were classified as “very near the rioting area”. Finally, any score between these two ends, is classified as “near the rioting area”. The second section consisted of questions and sub-dimensions aimed at assessing the effects on the biopsychosocial spheres (4 questions and 3 sub-dimensions); a total of 35 biopsychosocial symptoms are addressed in this section. The insomnia severity index (ISI) instrument was incorporated in the third section; this is a self-report questionnaire intended to assess the nature, severity and impact of insomnia (7 questions). This questionnaire has been validated in Spanish^23^.

The results obtained were interpreted based on a total score resulting from the sum of all the responses’ individual score; recorded results were divided into 4 categories, classified as follows: absence of clinical insomnia (0 to 7 points), subclinical insomnia (8 to 14 points), moderate clinical insomnia (15 to 21 points), and severe clinical insomnia (22 to 28 points). The fourth section incorporated the Epworth sleepiness scale (ESS), which is a simple questionnaire validated in Spanish^24^, aimed at assessing subjective daytime sleepiness in the context of sleep disorders (8 questions). This questionnaire evaluated the propensity to fall asleep in 8 different sedentary situations. Each item may have a score from 0 (never) to 3 (frequently), with a total score ranging from 0 to 24. Total score of 9 or lower is considered normal; 10 to 12 is an indication of mild sleepiness; and 13 or higher indicates severe sleepiness.

### Statistics

The collected data were analyzed using the Stata 14 statistical software. Specifically, we used the ZINB command for the zero inflated negative binomial estimation, which includes the alpha test for testing the overdispersion assumption and the Vuong test for testing the zero-inflated assumption. Collected data were turned to variables that could be subjected to an econometric analysis. Particularly, the symptoms (biological, psychological, and social) were quantified and answers to the questions from the ISI and ESS questionnaires were converted into one score per person. Finally, data were registered and the descriptive statistics and results of the analyses are presented below. A zero-inflated model is appropriate since nearly 10% of the observations are zero (zero ISI or Epworth scores). Moreover, a negative binomial model is appropriate taking into account that the variance is significantly larger than the mean (overdispersion). Both assumptions, zero-inflation and overdispersion are empirically tested. The marginal effects are additive, and statistical significance was considered at the 5% level.

## RESULTS

Table 1 shows the descriptive statistics of the variables captured in the questionnaire. The categorical variables were expressed as counts and frequencies, whilst continuous data were presented as mean (standard deviation). ISI and Epworth scores are used as dependent variables later on. Based on the ISI score, almost 71% of the sample presents some type of insomnia. According to Epworth score, nearly 50% of the sample is classified as affected by moderate or severe daytime sleepiness. In both cases, the higher the score, the stronger the symptoms. Of the 2,532 people who answered the survey, 70.8% of the sample are women, 76.9% are ≤40 years old and 69.3% have an occupation (study or work) in healthcare, and 83.4% have a professional or technical degree. In some extent, this population group is over-represented compared to the overall country. Rather than extrapolating the results of the sample to the general population, we think it is of informative value to understand how some determinants have a greater impact on the indicators of sleep and insomnia of the surveyed subjects. In addition, average values exceeded the normality cutoff point, both in daytime sleepiness and insomnia. More than 70% of the respondents uses 2 or more online media to get information; in contrast, more than 55% of the respondents uses 2 or more offline media. As a result, almost 46% of the sample are exposed to information 3 or more hours per day. Finally, 85% of the respondents stated that they live or work near or very near to a rioting area.

**Table 1:** Descriptive Statistics.

Variable	Obs	Mean	SD	Min	Max
ISI score	2,651	11.407	6.290	0	28
Epworth score	2,651	9.651	5.177	0	24
**Gender**					
male (%)	774	29.197			
female (%)	1,877	70.803			
**Age Group**					
30 or less (%)	1,125	42.437			
31 to 40 (%)	861	32.478			
41 to 50 (%)	370	13.957			
51 or more (%)	295	11.128			
**Education Category**					
Up to high school (%)	96	3.621			
Incomplete College (%)	108	4.074			
College/Technical Student (%)	235	8.865			
College/Technical Degree (%)	2,212	83.440			
**Employment Status**					
Inactive (%)	155	5.847			
Employer (%)	157	5.922			
Student (%)	315	11.882			
Worker (%)	1,462	55.149			
Independent (%)	562	21.200			
**Occupation health**					
No health related (%)	813	30.668			
Health related (%)	1,838	69.332			
**Number of Offline Info Sources**					
0 (%)	354	13.353			
1 (%)	792	29.876			
2 (%)	799	30.140			
3 (%)	507	19.125			
4 (%)	199	7.507			
**Number of Online Info Sources**					
0 (%)	167	6.300			
1 (%)	603	22.746			
2 (%)	832	31.384			
3 (%)	698	26.330			
4 (%)	351	13.240			
**Number of Hours News/day**					
0-1 hrs (%)	664	25.047			
2 hrs (%)	778	29.347			
3 hrs (%)	436	16.447			
4 or more hrs (%)	773	29.159			
**Closeness to Disturbed Area**					
Far away from disturbed area (%)	383	14.447			
Near to disturbed area (%)	1,645	62.052			
Very close to disturbed area (%)	623	23.501			

**Figure 1:**
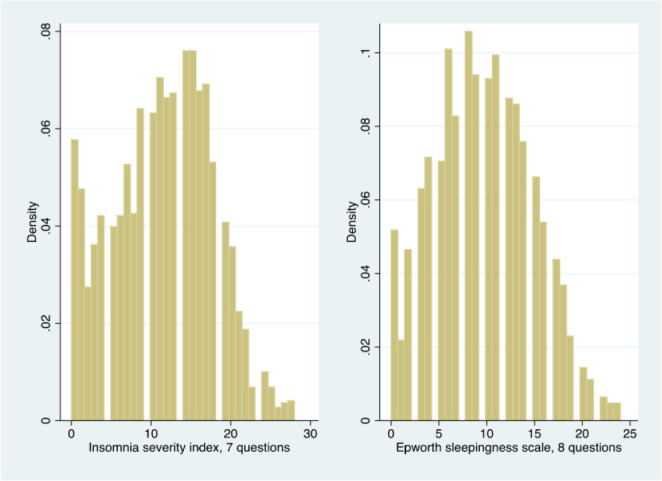
Histogram of the responses to the ISI and Epworth questionnaires.

To determine the effect of living/working near the rioting areas on the variance of the ISI and ESS scores independently from other predictors, we employed multivariate zero-inflated negative binomial models. Variables used as covariates were gender, age, education, employment status, health-related occupation, and information sources. Table 2 shows the marginal effects, at the average, after a zero-inflated negative binomial estimation. Epworth and ISI scores are driven by similar (Figure 1), but not the same determinants. Females aged 51 or older, who are neither studying a healthcare degree nor working in the healthcare sector, that are exposed to 4 or more hours per day to the news and live in areas near or very near the riots, have significantly higher scores. For instance, on average, females have 1.434 (SE 0.269, *p*-value 0.000) ISI score and 1.289 (SE 0.221, *p*-value 0.000) Epworth score more than males.

**Table 2:** Marginal Effects and Standard Errors from Two Multivariate Zero-Inflated Negative Binomial Models for the Outcomes of Insomnia and Daytime Sleepiness.

Variables	ISI Score	Epworth Score
Gender (0= male, 1= female)	1.434***	1.289***
	(0.269)	(0.220)
31 to 40 years old	0.522*	0.484**
	(0.309)	(0.250)
41 to 50 years old	0.362	0.579*
	(0.401)	(0.327)
51 years old or more	0.406	1.013***
	(0.451)	(0.382)
Incomplete College	0.598	-0.079
	(0.983)	(0.740)
College/Technical Student	0.692	0.107
	(0.967)	(0.727)
College/Technical Degree	-1.037	0.016
	(0.761)	(0.592)
Employer	-0.138	-0.348
	(0.786)	(0.594)
Student	-1.349	0.037
	(0.989)	(0.795)
Worker	-0.904	0.209
	(0.634)	(0.480)
Independent	-0.669	-0.084
	(0.668)	(0.507)
Health occupation (0=no, 1=yes)	-1.660***	-0.940***
	(0.294)	(0.235)
1 offline info source	-0.356	-0.116
	(0.398)	(0.323)
2 offline info sources	-0.176	0.148
	(0.395)	(0.322)
3 offline info sources	0.206	0.563
	(0.435)	(0.356)
4 offline info sources	0.534	0.271
	(0.565)	(0.449)
1 online info source	0.602	0.148
	(0.520)	(0.435)
2 online info sources	0.972*	0.386
	(0.511)	(0.427)
3 online info sources	1.426***	0.665
	(0.532)	(0.443)
4+ online info sources	1.946***	0.762
	(0.592)	(0.488)
2 hours of news/day	0.290	0.365
	(0.316)	(0.262)
3 hours of news/day	0.673*	0.012
	(0.375)	(0.302)
4+ hours of news/day	1.587***	0.903***
	(0.341)	(0.275)
Near to disturbed area	1.356***	0.693**
	(0.381)	(0.320)
Very close to disturbed area	2.952***	1.625***
	(0.448)	(0.367)
Observations	2,651	2,651

Note: Standard errors in parentheses.

*** p<0.01,

* 2,651* p<0.05,

* p<0.1. ISI and Epworth scores are the dependent, outcome variables. The independent variable of interest is the distance from the disturbs, and the rest of the predictors that are used as possible confounders such as, gender, education and media exposure related variables. The reference groups are omitted, which correspond to male, less than 30 years old, who finished high school, inactive, in no health-related occupation, no offline info source, no online info source, far away from a disturbed area. The alpha parameter (the dispersion parameter) is 0.154 [0.139, 0.170] for the ISI score estimation and 0.191 [0.175, 0.209] for the Epworth score estimation. In both estimations, the confidence interval for the alpha parameter does not contain zero, therefore, the variance is significantly larger than the mean. The significant results of the vuong test result (zero inflated negative binomial vs. standard negative binomial) shows a z-value of 7.55 (p-value 0.000) for the ISI scores estimation and 6.54 (p-value 0.000) for the Epworth score estimation. These results support that the high- zero proportion leads to different results, therefore, a zero-inflated model is appropriate to model the data.

However, ISI scores seem to be more affected by the use of online information sources while Epworth scores are more affected by offline information sources. In terms of scores, living/working very close to a disturbed area has the largest effect among other determinants. Moreover, the larger the number of hours of news watched/read per day, the larger the score effect. Neither education level nor employment status tend to have a significant effect on scores. Finally, with respect to the effect of the main variable of interest, respondents located near disturbed areas have 1.356 ISI score (SE 0.381, *p*-value 0.000) and 0.693 Epworth score (SE 0.320, *p*-value 0.030) more than responds living far away from disturbed areas, while this effect more than doubles in very close to disturbed areas.

**Supplementary Figure 1:**
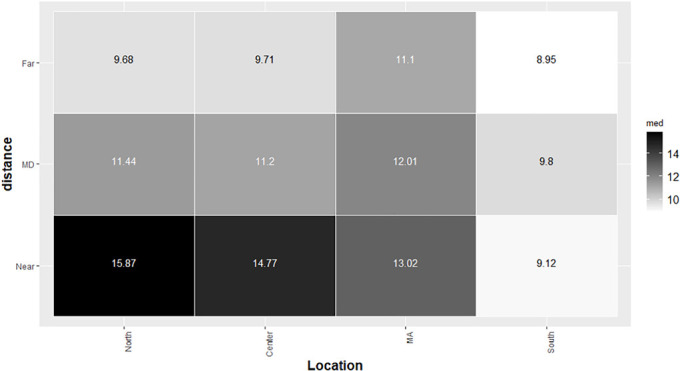
Average score of insomnia, based on the distance from the protests and riots and geographical location.

MA: Metropolitan Area Near: In the very same area of protests and riots (located in the same block, same street, streets intersection, adjacent or contiguous street); Medium nearness: Within a radius of 2 to 3 blocks away from the area of protests and riots (same neighborhood); Far: Very far from the area of protests and riots (another sector or commune).

**Supplementary Figure 2:**
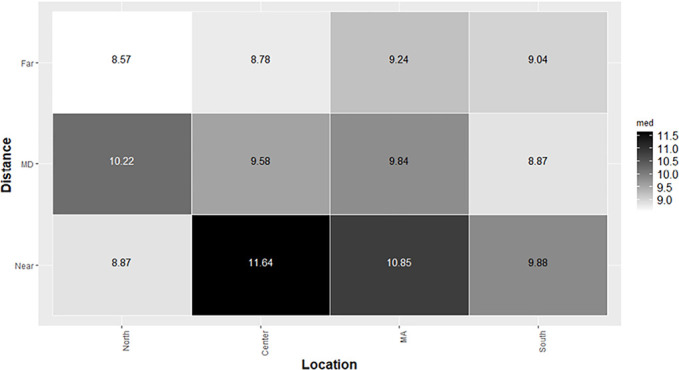
Average score of daytime sleepiness, based on the distance from the protests and riots and geographical location.

MA: Metropolitan Area Near: In the very same area of protests and riots (located in the same block, same street, streets intersection, adjacent or contiguous street); Medium nearness: Within a radius of 2 to 3 blocks away from the area of protests and riots (same neighborhood); Far: Very far from the area of protests and riots (another sector or commune).

Supplementary Figures 1 and 2 show that both insomnia and daytime sleepiness are greater the closer participants are to protests and riots, whether they live in the North, Central, Metropolitan, and South regions of Chile.

## DISCUSSION

The main result of the present study shows that the closer the person is living or working respect to the rioting spots in the city, the greater the possibility of suffering from insomnia and daytime sleepiness, regardless of age, gender and level of education.

In our study, 34.4% of the sample had insomnia (30% moderate and 4.4% severe, according to the ISI). These results are similar to those obtained in other studies, for instance, in a survey conducted in several countries such as France, Italy, Japan, and the United States, the estimated prevalence of insomnia ranged from 6.6% (France and Italy) to 37.2% (United States)^25^. Another recent study in Lebanon showed that 47.1% of the population suffers from insomnia. Among the factors associated with insomnia it is worth mentioning a death in the family during the last year, caffeine intake before going to bed, increased stress levels, depression, and anxiety^26^.

It is interesting to note that both insomnia and daytime sleepiness occur in a higher proportion in subjects who are presenting more than ten symptoms or negative biopsychosocial consequences. Probably, people who stay at home like housewives, unemployed people, elderly people, patients treated at home, or those people who have had to arrive earlier at their homes to get into their houses before the riots in the surrounding areas begin (and who are more exposed to witness the rioting or paying closer attention to the news), are the most affected, compared to people who live or work far from the public disturbances and who are less attentive to the news, especially on offline media. In the case of elderly and retired people, even the most active and those who participated in community groups, are likely to have been forced to suspend their community activities due to the protests or have had to lock themselves up and self-isolate in their homes to elude protests and riots in the city, which finally ends up exposing them more in their own home, and consequently, deteriorating their sleep quantity and/or quality.

The high levels of stress could be the mediators explaining sleep disturbances affecting people who live closer to the rioting areas; among the stressors that could be influencing the increase of insomnia and daytime sleepiness, we can mention the high psychosocial burden, the lack of control in working schedules, and insufficient rest^27-29^. It is also worth mentioning other possible factors that may be influencing the high levels of insomnia and daytime sleepiness, such as hormonal alterations, anxiety, and depression. The deregulation of the circadian cycle can lead to alterations of the hypothalamic-pituitary-adrenal axis (HPA). The HPA axis is a physiological system activated by stress, triggering the consequent release of adrenocorticotropic hormone (ACTH) from the pituitary gland and cortisol from the adrenal gland. Previous studies have suggested that people with poor sleep quality show a greater HPA axis stress response when exposed to physical and psychosocial stressing factors^30^. Other studies link sleep disorders with depression^31-36^ and various studies have associated sleep alterations with anxiety. A Chinese study showed a positive association between poor sleep quality and symptoms of anxiety; this association was significant among both males and females while the strongest positive associations were found among people aged 60 or older, smokers and people with a low level of physical activity, obesity and type 2 diabetes^37^. However, the mechanistic relationship between depression, anxiety, and sleep has not been completely understood yet, and has been considered complex and probably bidirectional^35,38^.

The news media have a potential impact on insomnia and daytime sleepiness, especially offline media, due to the distribution of information and images via WhatsApp or other social networks; these may probably be fake news or facts without journalistic editing, and may show shocking pictures or misleading information that might strongly affect people’s mood and increase their worries. Some studies have associated the time spent watching TV with a higher risk of insomnia^39,40^ and poor sleep quality; however, this seems to be the first study to show differentiated effects according to the type of media. The job search website Laborum.com4 presented the results of a contingency survey which purpose was to know the employees’ view regarding the situation in Chile; 1,476 people participated in the survey. It addressed some questions about the health of employed people. Employees were asked if the latest incidents had affected their mood and 81.4% assured that yes, they were indeed affected; some respondents said they had slept poorly (19.9%); others said they felt more nervous, irritable and sensitive (51.8%), and also they had experienced anxiety (9.7%); only 18.5% said they had not been affected at all^4^.

At international level, there is little evidence with respect to mass events and their effect on sleep, insomnia or daytime sleepiness. An Israeli study, that evaluated insomnia during the Persian Gulf War, showed that 10% complained of multiple awakenings during sleep, 4.5% mentioned they had difficulty falling asleep and 13.5% said they experienced a combination of the two situations described. Women complained significantly more than men, and people with a lower level of education complained significantly more than people with higher education. Only 3% of the sample reported using sleeping pills^41^.

In the United States, people exposed to dust from the World Trade Center (WTC) have multiple comorbidities that affect sleep, including obstructive sleep apnea, chronic rhinosinusitis, gastroesophageal reflux disease, post-traumatic stress disorder, and poor sleep quality. The 74.8% of the people had obstructive sleep apnea (OSA)^42^.

On the other hand, a prospective study examined the subjective quality of sleep and excessive daytime sleepiness before, during and after the deployment of German soldiers in Afghanistan (sleep quality was assessed with the Pittsburgh sleep quality index [PSQI] and daytime sleepiness with the ESS. Sleep quality and daytime sleepiness were affected during the pre-deployment training phase and remained at that level during the deployment phase, clearly indicating there is a need to pay more attention to sleep in young soldiers, even in this early stage. The percentage of poor sleepers decreased significantly after the deployment. Programs to teach techniques to improve sleep quality and stress management should be implemented prior to the deployment in order to reduce sleep difficulties and excessive daytime sleepiness and subsequent psychiatric disorders^43^. This raises the need for the implementation of preventive and promotional strategies to promote sleep hygiene practices during the less vigorous and intense periods of social unrest, in order to face potential crises that may continue over time with varying intensities.

Recently, insomnia has been identified as a disorder and as a symptom of psychiatric and medical disorders. Insomnia and anxiety often coexisted; especially when there is a clear overlap of symptoms^38^. An early morning awakening is considered a hallmark symptom of depression, and resolution of insomnia is a predictor of a favorable response to depression treatment^44^.

A study that evaluated post-traumatic stress in military personnel and veterans showed that there are genes that are dysregulated and that are associated with sleep, circadian function, and metabolism. In view of these results, the authors of the article suggest that there is an association with excessive daytime sleepiness^45^.

The results indicating high levels of insomnia and daytime sleepiness that we found are likely to have risen due to the abrupt end of the social outbreak and the almost immediate commencement of mandatory and voluntary quarantines due to the COVID-19 pandemic, which brought new stressors such as job loss, domestic violence, food insecurity, among other factors^46-49^. Therefore, it will be relevant to strengthen studies about the effects of social crises and outbreaks (when the concept of collective violence is involved) on sleep and daytime sleepiness, as well as addressing the impact on people’s sleep of health emergencies such as the COVID-19 pandemic and natural disasters that take place on the planet, to proactively generate preventive and promotional strategies, and intervention strategies developed by professionals.

Among the strengths of this study, we can mention we used internationally validated questionnaires that makes it possible to compare our results with other studies. Among the weaknesses, this is a cross-sectional study, so we cannot speak of causality but only of association; a low proportion of surveys was answered by men; and since the study was conducted online, some people are not eligible to participate such as people with lower incomes who do not have access to the internet and those who do not use social networks such as elderly people. Finally, other psychiatric/psychological factors, such as anxiety and depression, together with the social outbreak, could have a considerable impact and influence on the observed results. Future studies must include mental health assessment.

## CONCLUSION

Insomnia and daytime sleepiness more frequently affect people who live or work near the rioting spots in cities, regardless of age, gender, and level of education.

It is necessary for healthcare workers to be able to identify problems associated with sleep, provide sleep hygiene indications for the general population, and refer to certified sleep specialists when appropriate.
